# Development of Wrist Interface Based on Fully Actuated Coaxial Spherical Parallel Mechanism for Force Interaction

**DOI:** 10.3390/s21238073

**Published:** 2021-12-02

**Authors:** Jaeyong Lee, Hyungjoo Kim, Woosung Yang

**Affiliations:** 1 School of Robotics, Kwangwoon University, 20 Kwangwoon-ro, Nowon-gu, Seoul 01897, Korea; leejy93@kw.ac.kr; 2 Hyundai Motor Company, Crash Safety, Saimdang-ro 17-gil 116 101-1105, Seoul 01897, Korea; kimhjoo@hyundai.com

**Keywords:** force interaction device, wrist exoskeleton, parallel mechanism

## Abstract

To develop a wrist robotic exoskeleton-type interface (REI) for force interaction, it should have a suitable range of motion similar to human wrist activities of daily living, large torque output performance, and low moving parts inertia for dynamic motion response to cover the human behavior frequency. In this paper, a wrist REI based on a fully actuated coaxial spherical parallel mechanism (CSPM) is proposed to satisfy the aforementioned features. The fully actuated CSPM-based wrist REI (FC-WREI) has the characteristics of pure rotation similar to the human wrist, high torque output by parallel torque synthesis, and low moving parts inertia due to the base arrangement of the actuators. Due to the mechanical advantages and design optimization, the FC-WREI maximally provides torque as much as 56.49–130.43% of the maximum isometric torque of the human wrist, while providing a consistent range of motion to the human wrist without interference problem. Moreover, it is confirmed that the inertia of the FC-WREI is up to 5.35 times lower than similar devices. These advantages of the FC-WREI mean that the device is applicable to various fields of REIs for force interaction.

## 1. Introduction

One of the prominent areas of robotics is the concept of a robotic interface with physical interaction. Various robotic interfaces are being developed for a number of applications [1,2]. These robotic interfaces can be generally classified into an exoskeleton-type and an end-effector-based type [3]. The robotic exoskeleton-type interfaces (REIs) have been widely used since they can apply torque to specific joints and accurately record and monitor the motion of the target joint [4]. One of the most studied REIs is to aid treatment for physical dysfunction or muscular training in the upper extremity. This is because the function of the upper extremity plays a very important role in activities of daily living (ADL) [5]. To this end, various mechanisms have been proposed to the REIs for shoulder, elbows, and so on [6]. Additionally, various REIs for the wrist are under study.

In previous studies, several parallel mechanisms were preferred for high stiffness, precision, high torque output, and low moving parts inertia. The pneumatic seven degrees-of-freedom (DOF) master arm [7], MAHI-EXO II [8], and RiceWrist [9] are such parallel-type wrist REIs; however, these structures have several issues: (1) some actuators were attached to moving links, thus increasing the moving parts inertia; (2) workspace was limited due to existence of kinematic singularity; and (3) the joint center of the REI was sometimes misaligned with that of the human wrist [10]. Schiele et al. warned that the misalignment of such devices may cause discomfort, pain, and sometimes injury [11]. Even though soft-actuator-type mechanisms such as [12,13] might be one of the solutions, there are limitations in achieving accurate posture and torque. The wrist REIs based on serial mechanisms were used to resolve workspace and misalignment issues [4,10,14,15,16,17,18]. For example, Pehlivan et al. modified the parallel-type RiceWrist to the serial-type RiceWrist-S. This structure realized pure spherical rotation suitable for wrist motion and a wider range of motion (ROM) than the human body [4,10]. However, the serial wrist REIs still suffered from inertial forces from the actuators attached to moving links. Such inertial forces are significant in the agile motion of each joint. It also acts as a constraint on actuator selection, which determines the torque and velocity performance of each joint. Recently, even though a mechanism to lower the moving parts inertia by utilizing a differential transmission has been proposed [19], since relatively heavy actuator components are still on the moving parts, the effectiveness of the mechanism in reducing the inertia is limited. In addition, a wrist REI used for sensitive force interaction should be satisfied with the following requirements [20]: wide ROM similar with human wrist motion, high torque output performance, and high dynamic motion to cover the human behavior frequency.

In a previous study, a parallel wrist REI was designed applying an over-actuated coaxial spherical parallel mechanism (CSPM) [21]. It was verified that the device has wide ROM similar with human wrist motion due to the coaxial actuation, optimal design to avoid interference problem, and high torque output capability due to the parallel torque synthesis. However, the device still suffered from the actuator inertia placed on moving parts, and the control difficulty by the actuator redundancy.

Hence, fully actuated three DOF CSPM-based wrist REI (FC-WREI) is newly proposed to overcome the aforementioned issues. Basically, the FC-WREI inherits the advantages of the previous device as the followings [21,22,23]: pure rotational DOF without translation, wide ROM with unlimited rotation to the specific axis, and torque amplification in terms of the parallel torque synthesis. In addition, it has several more benefits over previous and similar devices [8,15,16,18,19,21]. Simple kinematic analysis and force control for the proposed device become possible by a fully actuated, completely parallel mechanism. Because all actuators of the FC-WREI are only installed on the fixed base, the weight of the actuators does not affect to the moving parts inertia. This design makes the FC-WREI have low moving parts inertia, which is advantageous for high dynamic motion response and efficiency on torque amplification. These advantages of the FC-WREI are suitable for developing the force interacting wrist REIs that can be applied to various devices for haptic, rehabilitation, exercise, tele-operation, and so on.

The remainder of this paper is organized as follows. A kinematic analysis of the FC-WREI is described in Section 2. The problem of interference is described in detail in Section 3. Multi-objective optimization through performance indices and its results are presented in Section 4. Section 5 details the procedures and results of simulation and experiments to verify the feature of the FC-WREI. Our conclusions and future work are discussed in Section 6.

## 2. Kinematic Analysis of the CSPM

**Figure 1:**
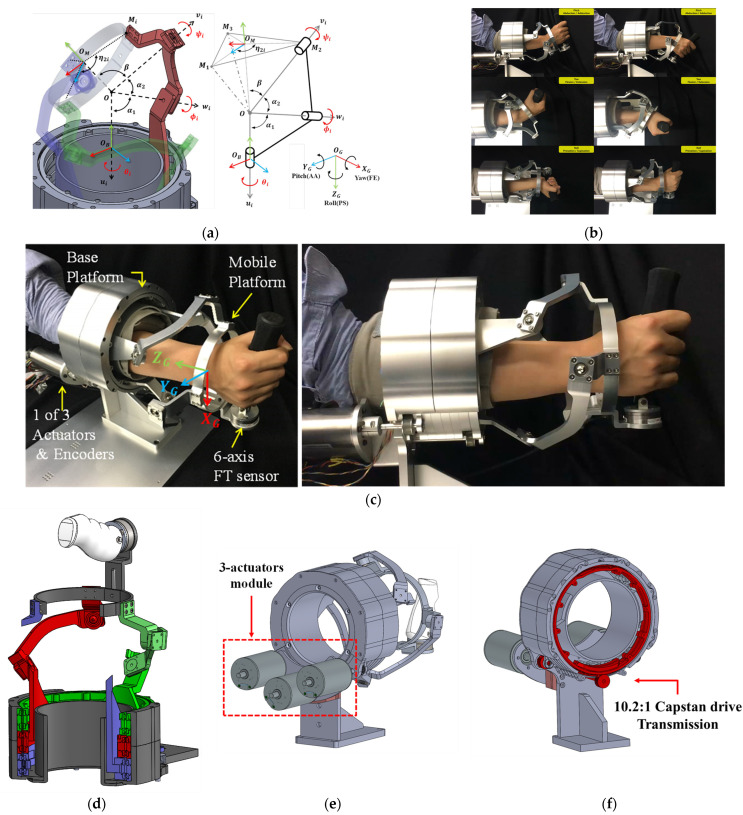
Overview of the FC-WREI: (**a**) represent the kinematic scheme and definition of the geometric design parameters, (**b**) shows some examples of limited poses with human, (**c**) shows the prototype design of the FC-WREI, and (**d**) shows a cross-sectional view. Three actuating slides moving on different tracks on the base. The details of the actuator placement and the transmission are represented in (**e**,**f**), respectively.

Generally, the spherical parallel mechanism consists of three serial arms, a base platform, and a mobile platform. The notations and symbols illustrated in Figure 1a are similar to those in [23]. Figure 1c shows the system prototype and how to use the FC-WREI. The subscripts B, M, and G denote the base, mobile platform, and global axes of the wrist, respectively. Each arm is composed of two serial links. The ith lower link is connected to the base platform through a revolute joint about the axis of $u_{i}$, with its rotation angle denoted by $\theta_{i}$. The upper link of each arm is connected to the mobile platform through a revolute joint about the axis of $v_{i}$, with its rotation angle denoted by $\psi_{i}$. Two links are connected through a revolute joint about the axis of $w_{i}$, with its rotation angle denoted by $\phi_{i}$. When the revolute axes of $u_{i},\;w_{i}$, and $v_{i}$ are expressed as global axes, $u_{i}\cdot w_{i}=cos(\alpha_{1})$ and $w_{i}\cdot v_{i}=cos\left(\alpha_{2}\right)$. The mobile platform imposes angle β between its normal vector and the revolute joint axes. The distribution of the three joints on the mobile platform with respect to the local *y* axis is denoted by $\eta_{2i}$.

### 2.1. Forward Kinematics

$u_{i}$ and $w_{i}$ are defined as

(1) $$u_{i}=R_{x}\left(-\pi /2\right)y_{0},$$

(2) $$w_{i}=R_{x}\left(-\pi /2\right)R_{y}\left(\theta_{i}\right)R_{x}\left(\alpha_{1}\right)y_{0},$$

where $y_{0}={\left[0\;1\;0\right]}^{T}$ is the *y* axis expressed using global axes and $R_{n}$ is a rotation matrix along the n axis. The rotation angles of passive joints are obtained through kinematic analysis of a 4-bar spherical linkage system. The passive angles of $\phi_{i}$ and $\psi_{i}$ are presented as a function of $\theta_{i}$ and design parameters. The input kinematics can be derived with Equation (3) via the half-tangent method with t=tan(ψ/2):

(3) $$\overset{8}{\underset{n=0}{\sum }}k_{n}t^{n}=0,$$

where $k_{n}$ is a function of the known design parameters. Thus, a maximum eight different kinematic solution exists. Since $\phi_{i}$ is a function of $\theta_{i}$ and $\psi_{i}$, the rotation matrix of the mobile platform is presented in terms of $\phi_{i}$ and $\psi_{i}$ as follows:

(4) $$R=R_{y}\left(\theta_{i}\right)R_{x}\left(\alpha_{1}\right)R_{y}\left(\phi_{i}\right)R_{x}\left(\alpha_{2}\right)R_{y}\left(\psi_{i}\right)R_{x}\left(\beta -\frac{\pi }{2}\right)R_{z}\left(-\eta_{2i}\right).$$

### 2.2. Inverse Kinematics

The rotation matrix R of the mobile platform can also be expressed in terms of Z-Y-X Euler angles, $\Phi_{roll}$, $\Theta_{pitch}$, and $\Psi_{yaw}$, which corresponding to pronation–supination (PS), adduction–abduction (AA), and flexion–extension (FE) motion, respectively. Let $v_{i}^{*}$ express $v_{i}$ represented in the mobile platform and $r_{nm}(n,m=1,2,3$) be the component of R at the nth row and mth column of the matrix. Then, $v_{i}$ is defined as $v_{i}=Rv_{i}^{*}$. Solutions to the inverse kinematics can be obtained using the relation of $w_{i}\cdot v_{i}=\cos\left(\alpha_{2}\right)$.

(5) $$\theta_{i}=2\;atan\left(\left(B_{i}\pm \sqrt{A_{i}^{2}+B_{i}^{2}-C_{i}^{2}}\right)/\left(A_{i}-C_{i}\right)\right).$$

where:

(6) $$s\left(\alpha \right)=sin\left(\alpha \right),\;c\left(\alpha \right)=cos\left(\alpha \right),\;A_{i}=s\left(\alpha_{1}\right)\left(r_{23}c\left(\beta \right)+r_{22}c\left(\eta_{2i}\right)s\left(\beta \right)-r_{21}s\left(\beta \right)s\left(\eta_{2i}\right)mma\right)rmrm),\;B_{i}=s\left(\alpha_{1}\right)\left(r_{13}c\left(\beta \right)+r_{12}c\left(\eta_{2i}\right)s\left(\beta \right)-r_{11}c\left(\beta \right)s\left(\eta_{2i}\right)\right),\;\;\;\;\;\;\;C_{i}=-c\left(\alpha_{1}\right)\left(r_{33}c\left(\beta \right)+r_{32}c\left(\eta_{2i}\right)s\left(\beta \right)-r_{31}s\left(\beta \right)s\left(\eta_{2i}\right)\right).$$

### 2.3. Jacobian Analysis

Since the SPMs do not have translational DOF, only the rotational Jacobian should be considered when deriving the Jacobian matrix. Angular velocity $\omega_{M}$ is the sum of the angular velocity vectors of each spherical arm.

(7) $$u_{i}{\dot{\theta }}_{i}+w_{i}{\dot{\phi }}_{i}+v_{i}{\dot{\psi }}_{i}=\omega_{M}.$$

Let $\dot{\Theta }={\left[\dot{\theta_{1}}\;\dot{\theta_{2}}\;\dot{\theta_{3}}\right]}^{T}$; then, Jacobian J can be expressed as $J=A^{-1}B$, from Equation (9), where A and B are presented in Equation (8) and diag denotes a diagonal matrix:

(8) $$A=\left[\begin{array}{c} {\left(w_{1}\times v_{1}\right)}^{T}\\ {\left(w_{2}\times v_{2}\right)}^{T}\\ {\left(w_{3}\times v_{3}\right)}^{T} \end{array}\right],\;B=diag\left(\left[\begin{array}{c} \left(w_{1}\times v_{1}\right)\cdot u_{1}\\ \left(w_{2}\times v_{2}\right)\cdot u_{2}\\ \left(w_{3}\times v_{3}\right)\cdot u_{3} \end{array}\right]\right),$$

(9) $$A\omega_{M}=B\dot{\Theta }.$$

## 3. Interference Safety of the C-WERI

**Figure 2:**
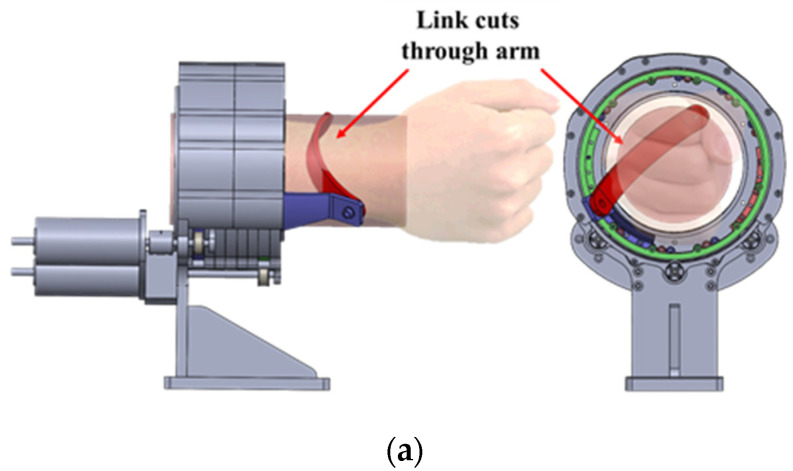

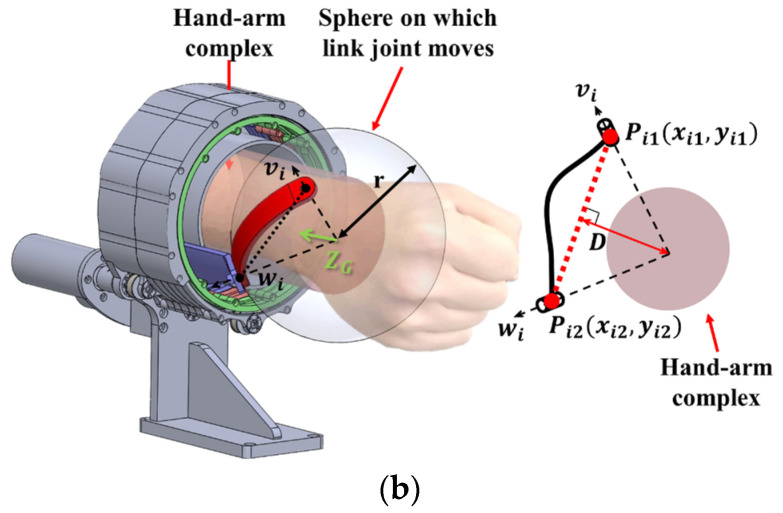
(**a**) An example situation of interference between the FC-WREI and forearm. (**b**) Description of D, which is a distance from a line between $P_{i1}$ and $P_{i2}$ to the centerline of the forearm.$\;P_{i1}\left(x_{i1},y_{i1}\right)$ and $P_{i2}\left(x_{i2},y_{i2}\right)$ are projections of the linkage joints on plane z=0.

In this paper, interference refers to the case in which links of the FC-WREI virtually cuts through the forearm during wrist motion; one such typical situation is illustrated in Figure 2a. Let the arm be represented by a cylinder with radius $r_{arm}$ and its centerline along the global Z-axis as $Z_{G}$. Let the radius of the FC-WREI sphere that the linkage joints float on be $r_{CSPM}$. Since $r_{CSPM}$ is larger than $r_{arm}$, interference only occurs when an upper linkage of FC-WREI passes through the cylinder. Therefore, this can be simplified and expressed on a projection plane. Since the interference condition is a complex function of kinematic design and wrist motion, iterative numerical evaluations are needed. The interference safety margin (ISM) $\bar{D}\;$ is defined as the minimum value of the linkage-to-arm distance during wrist motion over the workspace:

(10) $$\bar{D}\;\left(\Omega ,\;\kappa \right)=a\;minimum\;of\;D\left(\Phi ,\kappa \right),\;\Phi \;\in \;\Omega ,$$

where $\Phi =\left[\Phi_{roll},\Theta_{pitch},\Psi_{yaw}\right]\;$ is a vector for the orientation of a mobile platform; κ  is a set of kinematic design parameters of the FC-WREI consisting of $\alpha_{1}$, $\alpha_{2}$, β, $\eta_{21}$, and $\eta_{22}$; Ω is a set of wrist postures and comprises the workspace; and D(Φ,κ) is the distance from a line segment $\bar{P_{i1}P_{i2}}$ connecting two joints of an upper linkage to the centerline of the forearm at wrist posture Φ (Figure 2b), and is presented as in our previous work [21]. Let $\Delta x_{i}=x_{i2}-x_{i1}$ and $\Delta y_{i}=y_{i2}-y_{i1}$, then D(Φ,κ) is determined as follows:

(11) $$D\left(\Phi ,\;\kappa \right)=\frac{\left|y_{i2}-\left(\Delta y_{i}/\Delta x_{i}\right)x_{i1}\right|}{\sqrt{{\left(\Delta y_{i}/\Delta x_{i}\right)}^{2}+1}},$$

To avoid interference, the following condition must be satisfied:

(12) $$\bar{D}\;\left(\Omega ,\;\kappa \right)\geq r_{arm},$$

where $r_{arm}\;$ is the radius of the forearm. $r_{arm}\;$ of the prototype device is set to 50 mm, which is a 27.9% margin in addition to the general forearm radius of a Korean person [24]. Though a large $\bar{D}\;\left(\Omega ,\kappa \right)$ is preferred, there are tradeoffs between $\bar{D}\;\left(\Omega ,\kappa \right)$ and other performance measures. (1) A common method to consider to avoid interference is to scale up the design uniformly. However, the scaled-up design would increase the inertia of links, and its dynamic motion capability would degrade. A scale ratio of $r_{arm}/\bar{D}\;\left(\Omega ,\kappa \right)$ is a net value that achieves both noninterference and minimal degradation of dynamic motion capability. Note that since a net scale ratio has $\bar{D}\;\left(\Omega ,\kappa \right)$ as a denominator, a kinematic design with a larger $\bar{D}\;\left(\Omega ,\kappa \right)$ could achieve lower the link inertia. (2) Due to the spatial motion, a wider workspace range would result in a smaller $\bar{D}\;\left(\Omega ,\kappa \right)$. That is, we can increase the $\bar{D}\;\left(\Omega ,\kappa \right)$ by restricting the workspace range. However, a narrow workspace might cause discomfort to a human, since the wrist has quite a wide workspace range. Therefore, it is important to select an appropriate optimal design parameter.

## 4. Design Optimization

To cope with tradeoffs among some performance measures, a kinematic design that maximizes these measures is sought through optimization. It would be ideal to take all performance measures including workspace as objective functions for multi-objective optimization, but this technique would require a number of iterations to find a solution. Instead, we perform two optimization steps in this study, which we describe in detail in this section.

### 4.1. Design Parameters and Bounds

Design parameters $\alpha_{1}$, $\alpha_{2}$, β, and $\eta_{2i\;}$(*i =* 1, 2, 3) are selected to optimize the FC-WREI; $\eta_{23}$ is set to take the negative of $\eta_{22}$ (i.e., $\eta_{23}=-\eta_{22}$) to reduce the parameters of the optimization. The bounds of the design parameters are defined in Table 1.

### 4.2. Performance Measures

#### 4.2.1. Condition Index

Since the parallel robots mostly are good at motion/force transmission but not at dexterous manipulation [25], a global condition index (GCI) can be considered for optimization. The condition index has previously been adopted to the optimization by many researchers [26,27,28,29]. GCI is the average of local condition indices (LCIs) over the workspace. LCIs estimate kinematic performance of specific posture, in terms of accuracy, dexterity, and force isotropy, and take a value between 0 and 1. When the FC-WREI can generate entirely isotropic torque, LCI becomes 1 [29]. In addition, the closer the LCI is to 0, the closer the configuration is to the singularity. Therefore, it is possible to derive critical design parameters by maximizing overall LCIs to avoid singularities that may often happen in parallel robots. GCI and LCI are expressed as:

(13) $$\mathrm{LCI}\left(\Phi_{i}\right)=\frac{1}{\left\|J{\left(\Phi_{i}\right)}^{-1}\|\|J\left(\Phi_{i}\right)\right\|},$$

(14) $$\mathrm{GCI}=\frac{1}{n}\overset{n}{\underset{i=1}{\sum }}\mathrm{LCI}\left(\Phi_{i}\right),$$

where *n* is number of sample points of the workspace and the $\Phi_{i}$ represents the corresponding posture of the FC-WREI. ‖*J*‖ express the norm of matrix *J*.

#### 4.2.2. Interference Safety Margin

The interference safety margin $\bar{D}$ is the minimum distance between the links and the centerline of the forearm over wrist motion. During optimization, $\bar{D}\;$ is calculated on the normalized FC-WREI with $r_{CSPM}=1$. After optimization, the kinematic design is scaled by a factor of $r_{arm}/\bar{D}$, as explained in the previous section. To achieve the best design, the optimization should find a solution that maximizes $\bar{D}$.

### 4.3. Design Optimization

**Figure 3:**
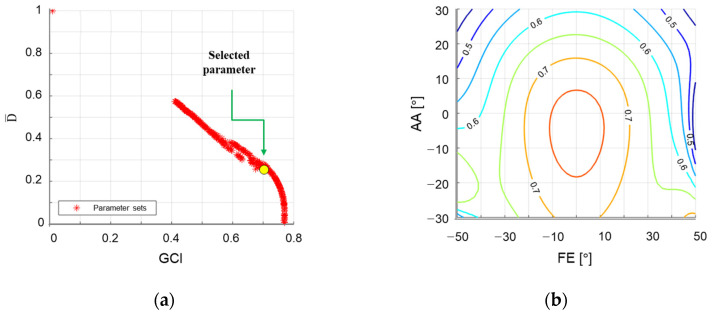
(**a**) The performance measures of the parameter sets after optimization. (**b**) Contour plot of LCI over the workspace. Since PS motion is independent of singularity, the contour plots are presented with respect to the other DOF. Each line is divided by 0.05. The minimum LCI over the workspace in (**b**) is 0.4.

**Figure 4:**
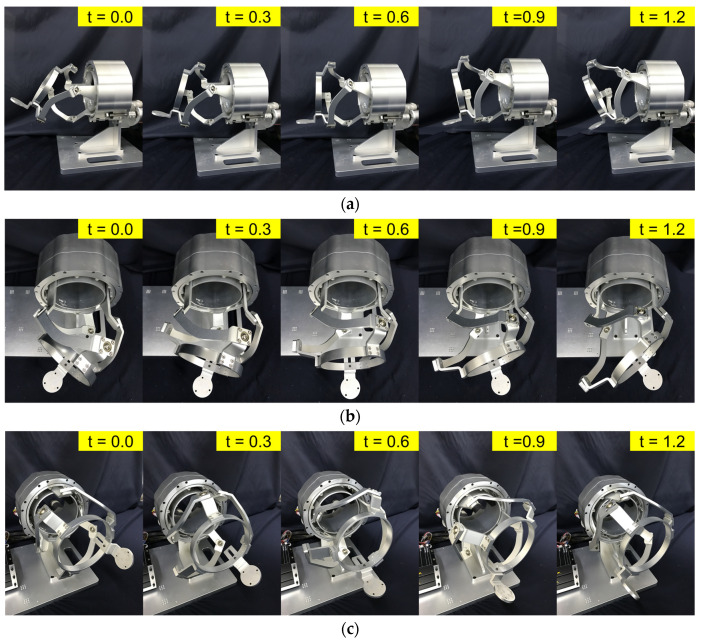
Design and motion example of the FC-WREI: (**a**) AA motion, (**b**) FE motion, (**c**) PS motion.

To maximize GCI and ISM, optimization is performed using non-dominated sorting genetic algorithm II (NSGA-II) [30]. We first solved preliminary optimization problems with different workspace scopes with the following conditions: an initial population of 300, a maximum of 200 iterations, a crossover probability of 0.9, a mutation probability of 0.2, and a distribution index of 20. The initial workspace scope was given as |PS| ≤ 80°, |FE| ≤ 70° and |AA| ≤ 60°. From this preliminary optimization, we found that the bounds of FE should restricted such that |FE| ≤ 50° and |AA| ≤ 30° to keep the LCI above 0.4 to ensure the minimum performance. The final optimization was carried out with an initial population of 100, 1000 iterations, a crossover probability of 0.9, a mutation probability of 0.2, and a distribution index of 20. The objective functions were imposed to maximize the GCI and $\bar{D}$, with inequality constraints of LCI > 0.4. The selected optimal design satisfied the aforementioned condition and also LCI > 0.7 within the prevalent workspace of |FE| ≤ 20° and |AA| ≤ 20°. Design parameters were rounded to one decimal place as $\alpha_{1}=57.5{}^{\circ}$, $\alpha_{2}=84{}^{\circ}$, *β* = 91°, $\eta_{21}=-3{}^{\circ}$, and $\eta_{22}=117{}^{\circ}$. The performance indices of the corresponding design are GCI = 0.701, and $\bar{D}\;$= 0.24. The final design achieves the ROM as |PS| ≤ 80°, |FE| ≤ 50° and |AA| ≤ 30° without singularities, which are 106.6%, 85.7% and 86.9% for the ADL ROM of the wrist. It is sufficient to entirely cover the functional ROM in [31]. Figure 3 shows the optimization results and LCI contour plot of the entire workspace and simple motion of the FC-WREI is described in Figure 4.

**Figure 5:**
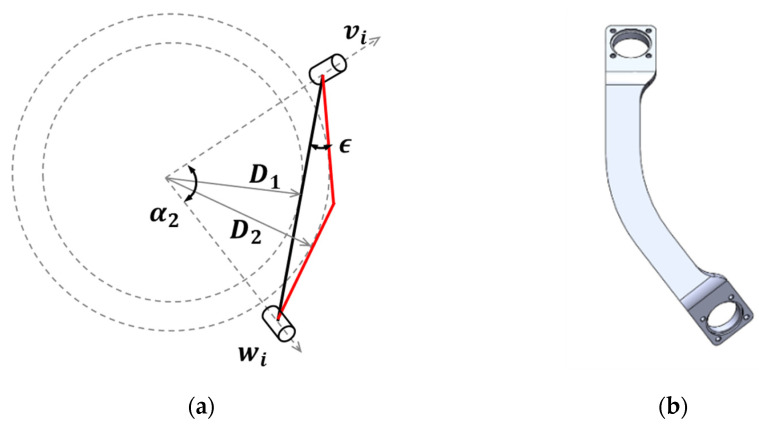
Description of the curved link: (**a**) the curved link (red) offers an increased ISM compared to the straight one (black) (*D*_2_ > *D*_1_), (**b**) the design of the curved link.

During optimization, interference safety margin $\bar{D}$ was calculated assuming that a link is straight. When the linkage is bent, $\bar{D}$ increases (Figure 5). Then, a scale ratio of $r_{arm}/\bar{D}$ for the FC-WREI decreases as explained in Section 3 so that the design becomes more compact and has low inertia. $\bar{D}$ increases from 0.24 to 0.43 when the bend angle ϵ of 27.9° is applied.

### 4.4. Actuator Capacity Design

Note that maximizing the condition index would lead to a design that not only increases isotropic torque performance but also avoids singularities. For the final FC-WREI design, the required actuator capacities are estimated by determining the maximum value of each actuator necessary to provide torque capability similar with the wrist torques of the ADL. The torque vector M is modeled as a function of two variables $\tau_{z}$ and ζ, the height and azimuth of a vector sphere, respectively, as follows:

(15) $$M={\left[\sqrt{1^{2}-\tau_{z}^{2}}\sin\left(\zeta \right),\sqrt{1^{2}-\tau_{z}^{2}}\cos\left(\zeta \right),\;\tau_{z}\right]}^{T},$$

where the values of $\tau_{z}$ and ζ are sampled evenly over $\left\{\left(\tau_{z},\zeta \right)|-1\leq \tau_{z}\leq 1,\;0\leq \zeta \leq 2\pi \right\}$. Joint torque of $\tau =J^{T}M$ is calculated repeatedly at each sample torque and sample wrist orientations within the ROM of the FC-WREI.

In the best configuration where the output torque is amplified by parallel driving, the largest required actuator torque is only 0.275 Nm to generate 1 Nm torque. This output torque amplification relative to the actuator is confirmed in 66.2% of the sample states for all postures and torque directions. At the worst sample states, the largest torque of the three actuators to generate the unit torque is 1.395 Nm. This indicates that the output torque to actuator torque is amplified up to 363.6%, although the worst becomes 71.6%.

The required actuator’s power capacities are estimated from the maximum actuator torques and their corresponding joint angular velocities. From Mann’s study [32], the maximum and average frequencies of the wrist motion during 24 prevalent ADLs were 2.47 Hz and 1 Hz, respectively. In this study, the target frequency is 5 Hz, which corresponds to a maximum of 31.4 rad/s for the mobile platform and 43.8 rad/s for the actuator based on the worst state in terms of velocity. In comparison with average torque output 2.47 Nm of similar devices [8,15,16,18,19], we selected the torque performance in all states to be at least 3 Nm to achieve sufficiently high torque output for various possible applications, e.g., not only rehabilitation but also haptic interface. Since the FC-WREI is the completely parallel mechanism, there are relatively few restrictions on the choice of actuator performance. Thus, the actuator capacity may be selected without the limitation of its weight according to the applications and design requirements. The required actuator torque was calculated as 4.18 Nm in the worst state. Assuming all the worst conditions, the required actuator power was calculated by multiplying the actuator torque of 4.18 Nm and the angular velocity of 43.8 rad/s. Then, the power requirement of each joint actuator was estimated to be about 180 W. The actuator system consisted of 180 W Maxon BLDC motor and wire transmission with a 10.2:1 reduction ratio. The rated torque of the motor is 0.419 Nm, and the weight is 0.823 kg.

## 5. Verification

### 5.1. Motion Control Experiment

**Figure 6:**
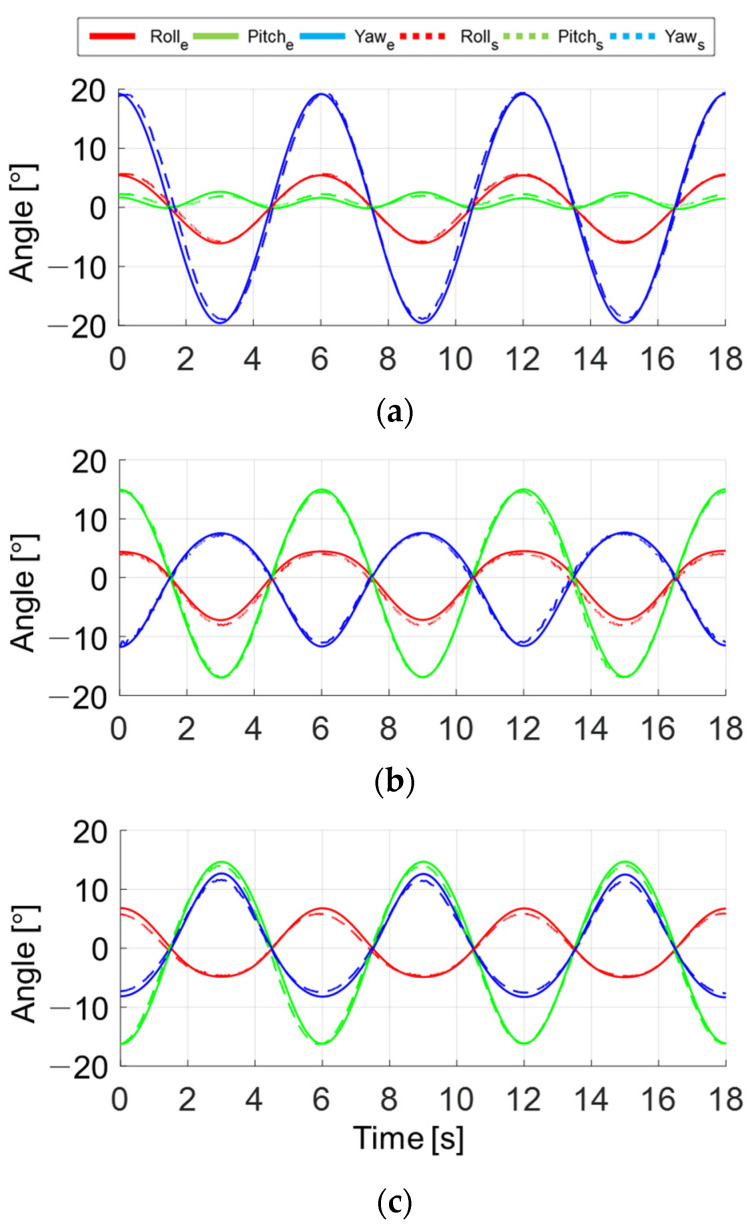
Results of the motion control experiment. The roll, pitch, and yaw denote the Euler angle of handle rotation. The subscript *e* and *s* denote the values measured and estimated from the kinematic model, respectively; (**a**–**c**) are the results for the joint *i* = 1, 2 and 3, respectively.

In a motion control experiment, the prototype is self-driven with joint PD control. The joint PD controller is constructed as $\tau =K_{p}\left(\Theta_{d}-\Theta_{c}\right)-K_{d}\left(\dot{\Theta_{c}}\right)$, where $\Theta_{d}$ is the desired angle, $\Theta_{c}$ is the current angle, and $K_{p}$ and $K_{d}$ are set at 400 and 40 respectively. Each actuator is controlled to make a sinusoidal motion with an amplitude of 18° and a period of 6 s while the other two actuators are kept static. An attitude and heading reference system (AHRS) installed at a handle measures the global Euler rotation angles. The model of the AHRS is MW-AHRSv1 manufactured by NTREX Corp. The static accuracy and resolution of the AHRS are 0.2° and 0.01°, respectively. The AHRS-based Euler rotation angles are compared with those converted from actuator angles using a kinematic model to verify the analysis. The results of the test for each actuator are shown in Figure 6a–c. Maximum average errors between measured and estimated angles for each test were calculated as 0.31°, 0.24° and 0.33°, respectively. Peak discrepancies for all tests were less than 1.04°. From the result, the kinematic analysis was verified through the small kinematic error. Since no external load was applied, the remaining kinematic error can be assumed to be manufacturing clearance. It should be improved in future versions of the FC-WREI.

### 5.2. Force Control Experiment

In this verification, a force control experiment with user is performed in static motion. A simple angle controlled one DOF device using the same actuator is applied as a user. Due to the fully actuated mechanism, general and simple force control can be applied to the FC-WREI. A simple impedance controller is constructed, and the admittance equations are designed as follows:

(16) $$I_{u}{\ddot{\Phi }}_{u}+B_{u}{\dot{\Phi }}_{u}+K_{u}\Phi_{u}=\tau_{u},$$

(17) $$I_{v}{\ddot{\Phi }}_{v}+B_{v}{\dot{\Phi }}_{v}+K_{v}\Phi_{v}=\tau_{v},$$

where $\Phi_{u}$ is the user intended motion, $\tau_{u}$ is the exerted torque about the wrist joint center, $\Phi_{v}$ is the virtual motion, $\tau_{v}$ is the virtual torque, respectively. The gains $I_{u}$, $B_{u}$, and $K_{u}$ are selected to satisfy critical damped condition with high stiffness such as $I_{u}=1$, $B_{u}=2\sqrt{K_{u}}$, and $K_{u}=10^{4}$, and $I_{v}$, $B_{v}$, and $K_{v}$ are set same as $I_{u}$, $B_{u}$, and $K_{u}.$ Desired motion $\Phi_{d}$ is expressed as $\Phi_{d}=\Phi_{u}-\Phi_{v}$ and the desired joint angles are calculated via inverse kinematics. $\tau_{u}$ was measured with the Mini40 6-axis FT sensor (ATI Industrial Automation) attached at the base of the handle.

**Figure 7:**
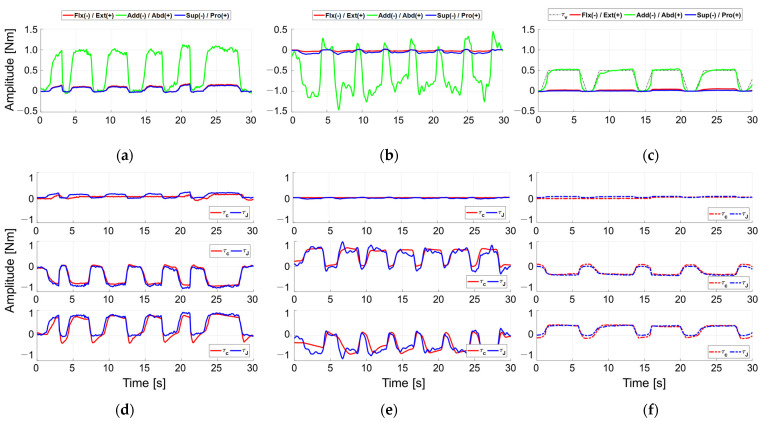
Measured torques during force control experiments for AA motion. (**a**–**c**) show the measured torque $\tau_{u}$ and (**d**–**f**) show the torques of three actuators from measurement (red) and those estimated from active currents in the actuators (blue). (**a**,**b**,**d**,**e**) are the control results according to the user’s intention without virtual torque, and (**c**,**f**) are the control results by the applied virtual torque while the user maintains the robot in its initial posture.

Figure 7 shows a case of the experiment for AA motion. Figure 7a,b show the measured wrist torque $\tau_{u}$ exerted by user intention while the device maintains the initial posture, $\Phi_{d}=0_{3\times 1}$. Figure 7c shows the measured $\tau_{u}$ controlled by virtual torque $\tau_{v}=0.5\;\mathrm{Nm}$, while the user maintains the initial robot posture. The actuator loads were calculated from those measurements in two ways: (1) the FT sensor measurement of torques and forces at the handle were first transformed into the $\tau_{u}$, and then converted into actuator loads $\tau_{J}$ through the relationship $\tau_{J}=J^{T}\tau_{u}$; or (2) the active currents of the actuators were converted into the exerted actuator torques $\tau_{c}$ using the electromagnetic relationship $\tau_{c}=nK_{t}i$, where n is a reduction ratio, $K_{t}$ is a torque constant provided by the manufacturer, and  i  is the measured active currents. The $\tau_{J}$ and the $\tau_{c}$ were logged during the tests on each axis. Red, green, and blue lines are the torque of direction of $X_{G}$, $Y_{G}$, and $Z_{G}$, as described in Figure 1c. Figure 7d–f show the actuator torques $\tau_{J}$ and $\tau_{c}$, order of the joint *i* = 1, 2, and 3 from the top. $\tau_{J}$ and $\tau_{c}\;$ are in good agreement, and discrepancy can be assumed to be due to the friction, complex force of the other axes, joint stiffness, and so on. The results present the feasibility of torque amplification, which is the feature of the FC-WREI. From the results of Figure 7c–d, joints 2 and 3 are mainly operated to generate AA directional torque. The peak end-effector torque to the actuator torque for each joint were calculated as 1.14 and 1.12, respectively. It means that the end-effector torque has been minimum 12% amplified from the single actuator torque. The maximum end-effector torque to the actuator torque for FE and PS motion were also calculated as about 0.99 and 2.86. Although some posture and end-effector torque direction may lead the amplification ratio under 1, it has been verified that the amplification effect by FC-WREI occurs in 66.2% of all postures and torque directions. Furthermore, the end-effector torque is amplified up to 3.63 times for a single actuator torque. Consequently, this feature widens the torque output with respect to the unit actuator or increases the ratio of output torque per unit actuator mass. This characteristic leads the FC-WREI to have high torque output capability. The maximum isometric torques of the FC-WREI prototype were also measured by the static force control experiment and are described in Table 2.

### 5.3. Dynamic Response Analysis

**Figure 8:**
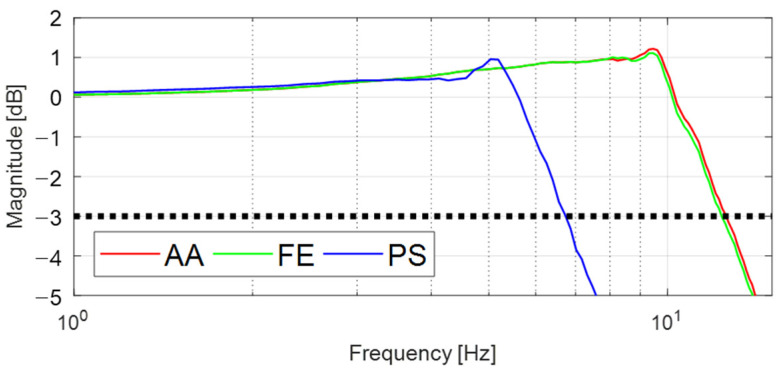
Frequency responses of the proposed device for each DOF from simulation.

**Figure 9:**
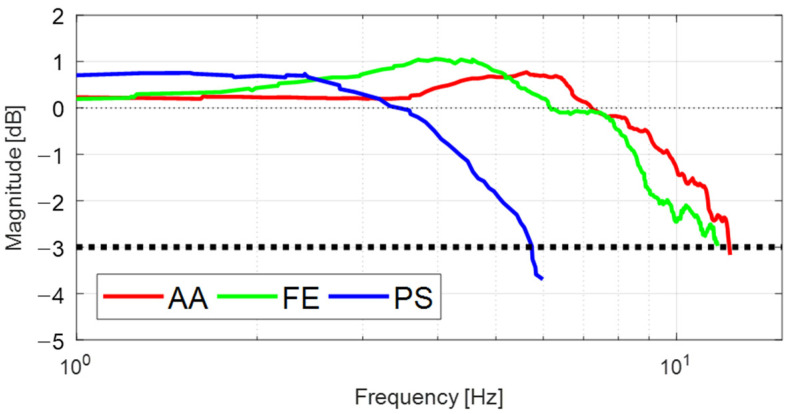
Frequency responses of the proposed device for each DOF from experiment.

To identify the dynamic response of the FC-WREI, a dynamic simulation using the MATLAB/SimMechanics toolbox was performed. The properties of the simulation model were derived from the prototype design. To analyze the characteristics for AA, FE, and PS motion, virtual massless joints were constructed at the origin of rotation and connected to the end-effector. From the initial configuration where the AA, FE, and PS angles are zero, a joint PD control simulation was performed for each virtual joint that tracks a swept sine signal with a magnitude of 10° and a frequency that varies from 0.1 to 15 Hz. The PD control gains were set high to have a bandwidth of 200 Hz and a phase margin of 85°. The closed-loop bandwidth frequencies (CLBW) for the AA, FE, and PS axes were measured as 12.51, 12.36 and 6.71 Hz, respectively (see Figure 8). Dynamic response analysis using the FC-WREI prototype was also performed with swept sine excitation. Figure 9 shows the frequency responses of the FC-WREI. The CLBW of the FC-WREI for the AA, FE, and PS axes indicate 11.83, 12.24 and 5.73 Hz, respectively. Although a slight discrepancy between the real and simulation models seems to be caused by mechanical properties of the prototype such as mechanical frictions, connecting joints, wire tension, and so on, it is confirmed that these bandwidth frequencies are larger than the target value of 5 Hz mentioned in Section 4.

**Figure 10:**
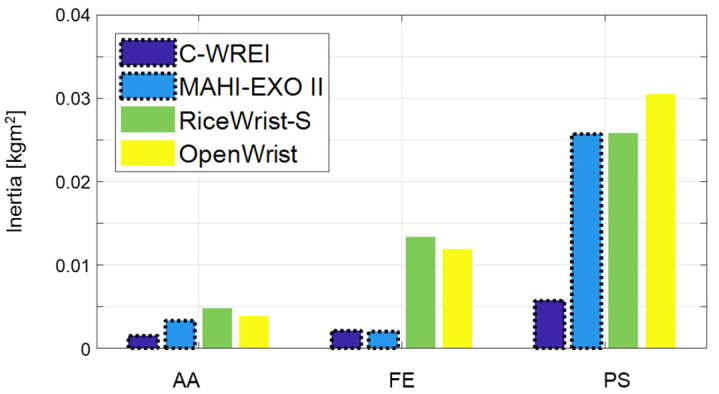
The estimated axial inertia of the wrist REIs. Bars with dotted lines show the inertia of the parallel mechanism-based wrist REIs including the FC-WREI.

Moreover, the inertia properties of each DOF were estimated adopting a logarithmic decrement techniques [34]. A virtual spring is implemented as $\tau_{\Phi }=K_{\phi }\left(\Phi_{d}-\Phi_{c}\right)$, where $K_{\phi }$ is set to be 1, 5 and 10; $\tau_{\Phi }$ is virtual control torque; and $\Phi_{d}$ and $\Phi_{c}$ are desired and current angle, respectively. The desired angle is set to be magnitude of 20° while the other two DOFs are constrained. From the vibrational response, mechanical natural frequency and the inertia properties are simply estimated. The estimated maximum inertia values for the AA, FE, and PS axes were 0.0015, 0.0021 and 0.0057 kgm^2^, respectively. Remarkable differences of the axial inertia with other similar REIs are revealed in Figure 10. It is confirmed that the FC-WREI has fairly low inertia values, which are up to 5.35 times lower than other similar REIs (compared to OpenWrist). Note that the FC-WREI has also relatively uniform inertia values for all DOF. The ratio between the maximum and minimum inertia of the FC-WREI is only 3.73, while the ratio of the other REIs is up to 8.02. These features lower the inertial forces that unintentionally occur in dynamic behavior, enabling the FC-WREI to operate dynamically and isotropically for any DOF. This is because the weight of the actuators does not affect the inertia of the FC-WREI. The similar REIs have large inertia due to the weight of the actuators placed on the moving parts. A comparison of dynamic characteristics of the FC-WREI and similar devices is presented in Table 3.

## 6. Conclusions

In this study, a fully actuated coaxial spherical parallel mechanism based wrist robotic exoskeleton-type interface was developed to satisfy wide ROM similar to a human wrist, with high torque output performance and low moving parts inertia for dynamic motion response. Based on the simple analysis of the fully actuated coaxial spherical parallel mechanism, the design parameters of the FC-WREI were optimized through the multi-objective optimization to increase kinematic performances as well as to reduce the inertia of the links while eliminating the possibility of colliding with the user. The FC-WREI realized the workspace scope of 85.7–106.6% of the ADL range of the wrist, while entirely covers the functional ROM. The workspaces for AA, FE, and PS DOF of the FC-WREI were ±30°, ±50° and ±80°, respectively. The ISM was introduced and maximized to 0.43 through optimization and further modification of the linkage curvature. By maximizing the ISM, the FC-WREI can be designed as compact to have low link inertia without interference problems. The optimal design also has the GCI of 0.701, while maintaining a local condition index greater than 0.4 throughout the workspace. By maximizing the GCI, the FC-WREI has the advantage of generating relatively isotropic torque even at extreme configurations near the workspace boundary. This makes it possible to give an appropriate torque output in any configuration in the workspace with high torque output performance which entirely covers the ADL torque and 56.49–130.43% of the maximum isometric torque of the human wrist. Moreover, the FC-WREI has low moving parts inertia by installing all actuators to the fixed base. This makes it possible to choose the actuators for the requirements of the various applications, as well as contributes to the high torque output performance of the FC-WREI by selection of the heavy and high torque actuator. The characteristics from the low inertia of the FC-WREI was verified through the dynamic response characterization. The closed-loop bandwidth frequency of the FC-WREI was 5.42–11.83 Hz, sufficiently high compared with the human wrist ADL frequency. It was also confirmed that the FC-WREI had fairly low inertia (0.0015–0.0057 kgm^2^) for all axes. This is up to 5.35 times lower than similar devices referred in this paper. These features indicate that the FC-WREI can be applied in various applications of the wrist robotic exoskeleton-type interfaces for force interaction, such as wrist muscle training and rehabilitation for physical function recovery, agile force and motion interface for tele-operation and haptic robots, and so on.

To accommodate the practical application of the proposed device, several issues must be resolved in subsequent studies. The acceptability of a ring-shaped exoskeleton-type device by patients should be tested, since the device traditionally causes aversion related to the fear of being unable to perform emergency evasion [35]. Moreover, evaluation of the FC-WREI in terms of the force interaction interface by user-test should be investigated in following studies.

## Figures and Tables

**Table 1:** The upper and lower bounds of the design parameters.

**Parameters (°)**	$\alpha_{1}$	$\alpha_{2}$	β	$\eta_{21}$	$\eta_{22}$
Upper bound	90	120	120	−30	150
Lower bound	30	60	60	30	90

**Table 2:** Comparison of the ROM and torque capability of similar wrist REIs. The table shows activities of daily living (ADL) and functional ROMs, the isometric torque of the human wrist [8,31,33], and the maximum continuous torque of the wrist REIs.

Wrist Motion	Pronation–Supination (PS)		Flexion–Extension (FE)		Adduction–Abduction (AA)	
	ROM (°)	Torque (Nm)	ROM (°)	Torque (Nm)	ROM (°)	Torque (Nm)
ADL	150	0.06	115	0.35	70	0.35
Functional ROM and Isometric torque	-	9.1	80	19.8	40	20.8
FC-WREI	160	11.87	100	14.34	60	11.75
MAHI EXO-II [8]	180	2.3	72	1.67	72	1.93
RiceWrist-S [8]	180	1.69	130	3.37	75	2.11
OpenWrist [18]	170	3.5	135	3.6	75	2.3
Wrist gimbal [15]	180	2.87	180	1.77	60	1.77
IIT wrist robot [16]	160	2.77	144	1.53	72	1.63
WRES [19]	140	6.52	75	1.62	40	1.62

**Table 3:** Closed-loop bandwidth frequency and inertia properties of the similar wrist REIs.

	FC-WREI		MAHI-EXO II		RiceWrist-S		OpenWrist	
DOF	CLBW (Hz)	Inertia (kgm^2^)	CLBW (Hz)	Inertia (kgm^2^)	CLBW (Hz)	Inertia (kgm^2^)	CLBW (Hz)	Inertia (kgm^2^)
AA	11.83	0.0015	10.6	0.0033	8.3	0.0048	9.8	0.0038
FE	12.24	0.0021	13.3	0.002	6.0	0.0134	7.0	0.0119
PS	5.73	0.0057	4.2	0.0257	3.5	0.0258	4.6	0.0305

## Data Availability

This paper did not generate research data to share.
